# Supplementary material for: A prefrontal-thalamic circuit encodes social information for social recognition
Source: Nat Commun. 2024 Feb 3;15:1036. doi: 10.1038/s41467-024-45376-y (PMC10838311; doi:10.1038/s41467-024-45376-y)
Supplement: Supplementary file 1 — Supplementary Information [file 41467_2024_45376_MOESM1_ESM.pdf]

Figure S1

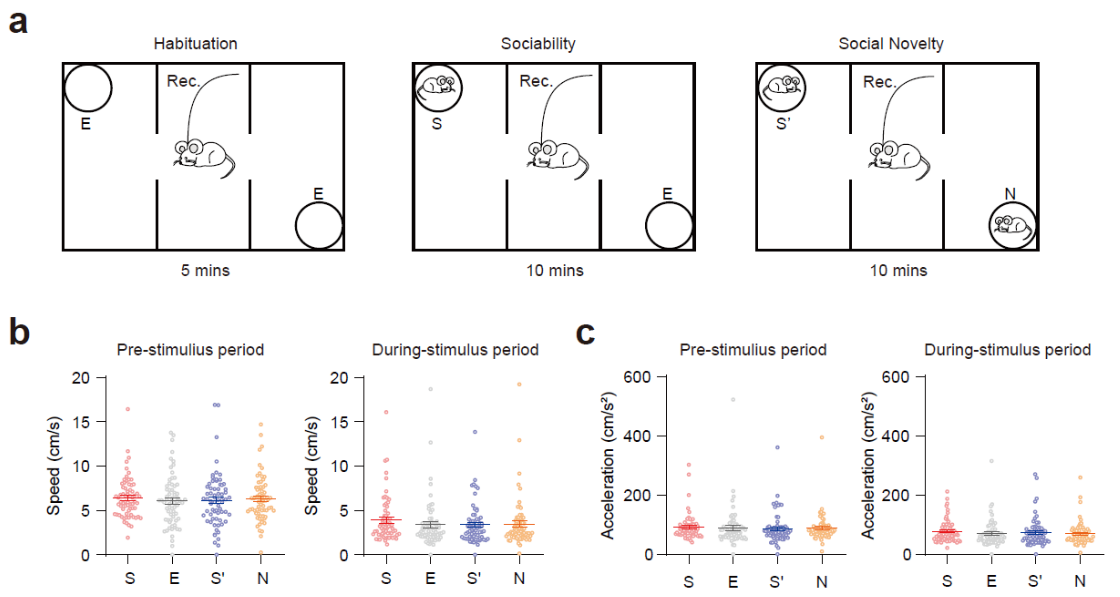

Supplementary Figure 1. **Motion measurements of the mice during the investigation of stimuli**

a, Three-chamber social interaction test consisting of habituation, sociability, and social novelty phases. The subject mouse was implanted with the electrode array and *in vivo* electrophysiological recordings were performed during the behavioral test.

b, c, Speed (**b**) and acceleration (**c**) when the subject mouse approached the stimuli (pre-stimulus period) and when it investigated the stimuli (during-stimulus period). The measurements were averaged across recording sessions. Data are mean ± s.e.m. Details of the statistical information are provided in Supplementary Data 1.

Figure S2

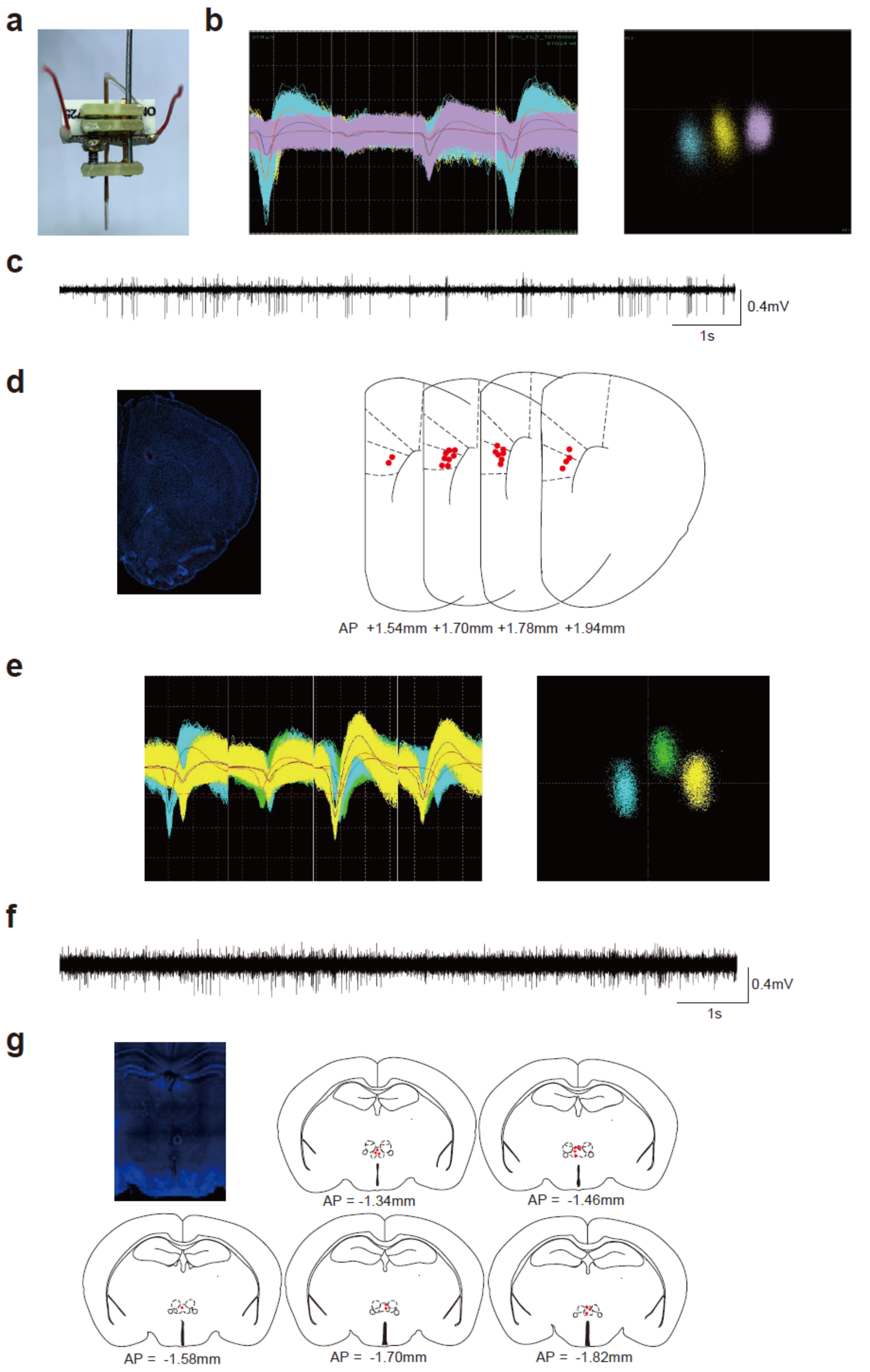

Supplementary Figure 2. In vivo single-unit recordings in mPFC and Re

a, The movable multiple electrode array consisting of tetrodes for single unit recordings.

b, Examples of spike waveforms (left) and the separation of single units in the principal component space (right).

c, Example of bandpass continuous data for spike recordings in the mPFC.

d, Location of the electrode by electrolytic lesion in the mPFC. The schematic drawing shows the locations of the electrode tips at the last advancement of the tetrode.

e-g, The same as **b-d** but for the Re area. The coronal brain atlas in (**b**, **d**) was produced based on Allen Brain Atlas from https://atlas.brain-map.org/.

Figure S3

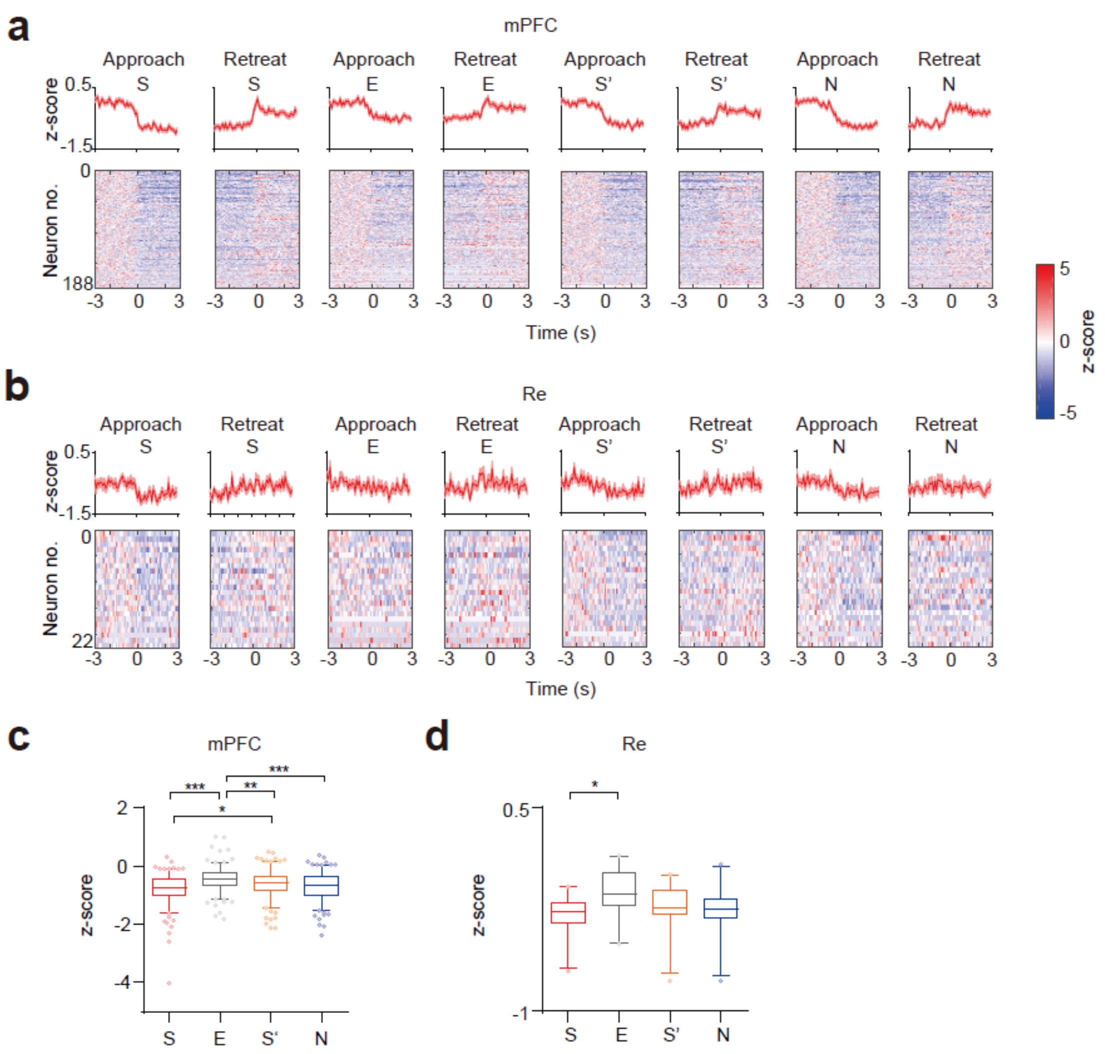

Supplementary Figure 3. mPFC and Re neurons with suppressed response

a, b, Response profiles of suppressed neurons to S, E, S' and N aligned at the beginning (approach) and the end (retreat) of the investigation.

c, d, Average response for each stimulus for mPFC and Re. Kruskal-Wallis test with Dunn's multiple comparison correction. **c,** n = 188 sessions for S, E, S', N. $H(3) = 44.15$. $P < 0.0001$. **d,** n = 22 sessions for S, E, S', N. $H(3) = 10.62$. $P = 0.014$.

*$P < 0.05$, **$P < 0.01$, ***$P < 0.001$. Box plots showing median, 25%-75% percentile, 5%-95% range and outliers. Line and shaded areas are mean ± s.e.m. Details of the statistical information are provided in Supplementary Data 1.

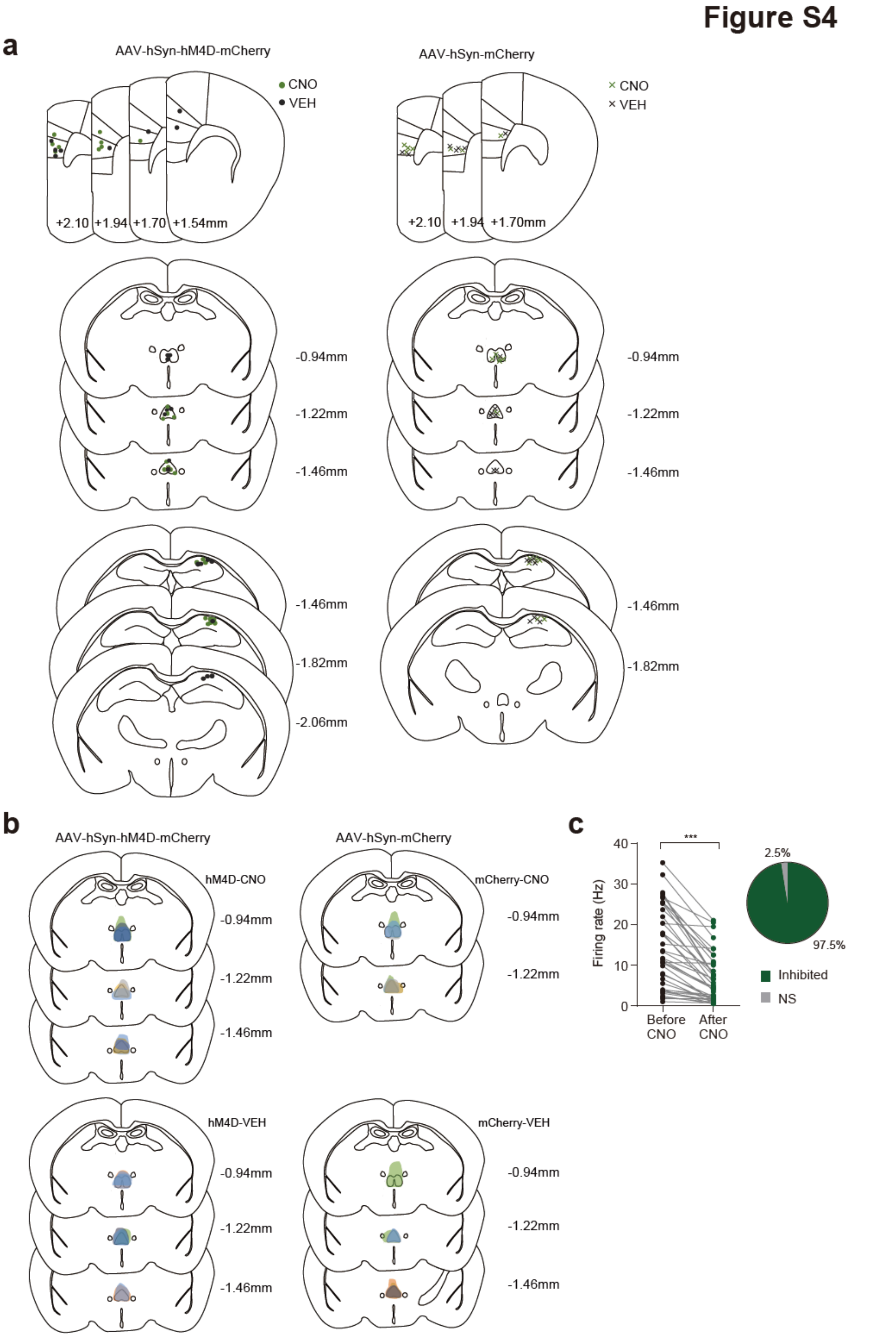

Supplementary Figure 4. Electrode locations and chemogenetic DREADD virus expression

a, Locations for electrode tips for local field potential recordings in mPFC, Re, and dHPC in mice infected with AAV-hSyn-hM4D-mCherry and AAV-hSyn-mCherry.

b, AAV-hSyn-hM4D-mCherry and AAV-hSyn-mCherry expressions in the Re. Each colored area corresponds to the virus expression in one mouse.

c, Firing rates of single-unit activities recorded by *in vivo* tetrode recordings before and after CNO injections. Pie graph showing the proportion of neurons that were inhibited. Comparison was performed by Wilcoxon matched-pairs signed rank test. $n = 40$ neurons.

$P < 0.0001$. NS, non-significant. ***$P < 0.001$. Details of the statistical information are provided in Supplementary Data 1. The coronal brain atlas in (**a**, **b**) was produced based on Allen Brain Atlas from https://atlas.brain-map.org/.

Figure S5

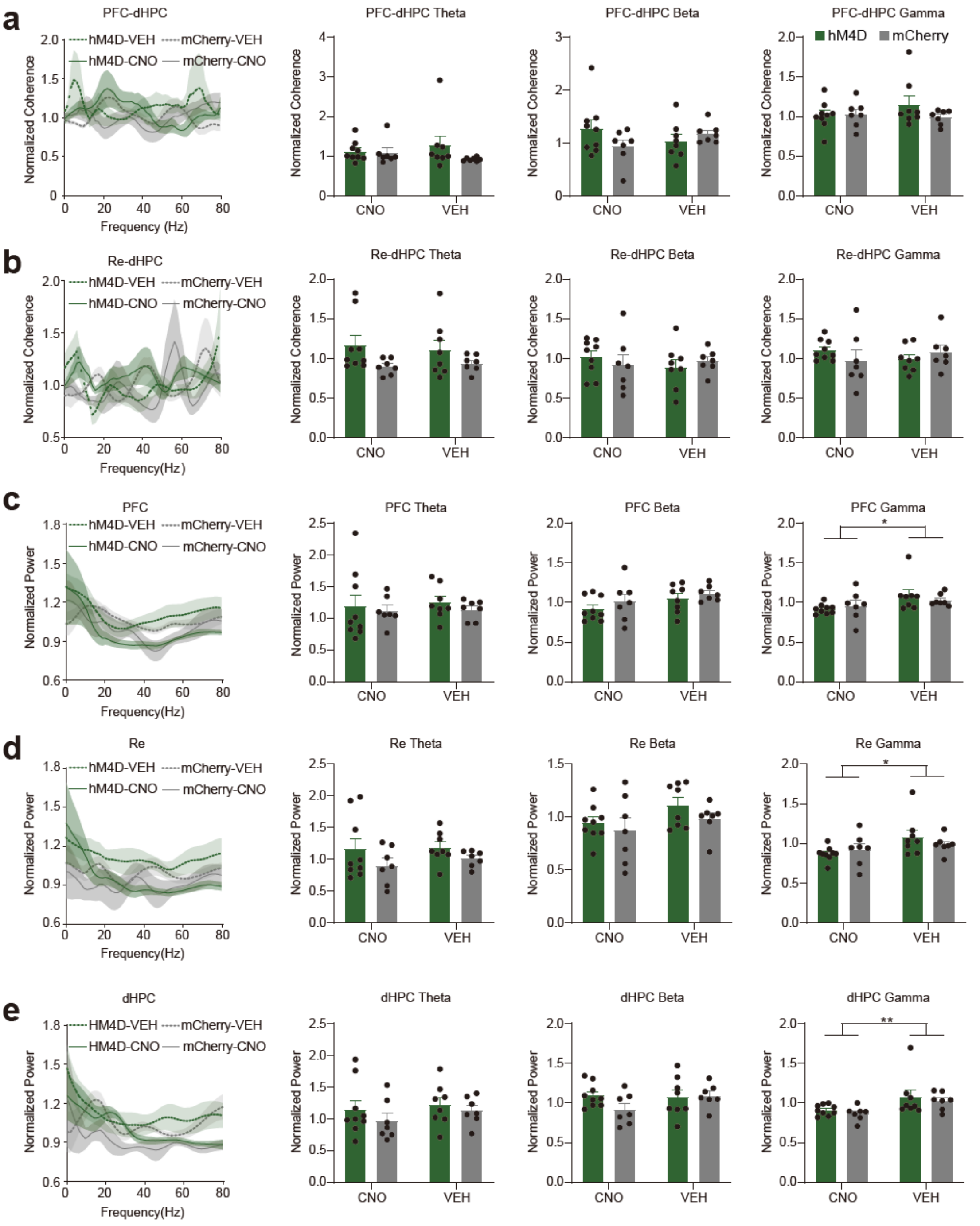

Supplementary Figure 5. Power and coherence analysis during chemogenetic inhibitions of the Re

a, b, Coherence under the treatment of CNO or VEH in mice infected with AAV-hSyn-hM4D-mCherry or AAV-hSyn-mCherry. **a**, PFC-dHPC coherence. **b**, Re-dHPC coherence.

c-e, Power under the treatment of CNO or VEH in mice infected with AAV-hSyn-hM4D-

mCherry and AAV-hSyn-mCherry. hM4D group, VEH: $n = 8$ mice. CNO: $n = 9$ mice. mCherry group, VEH: $n = 7$ mice. CNO: $n = 7$ mice. **c**, gamma power: treatment $F(1, 27) = 5.361$, $P = 0.0284$. **d**, gamma power: treatment $F(1, 27) = 4.789$, $P = 0.0375$. **e**, gamma power: treatment $F(1, 27) = 8.339$, $P = 0.0076$.

Two-way ANOVA. $^{*}P < 0.05$. $^{**}P < 0.01$. Line and shaded areas and bar graphs are mean ± s.e.m. Details of the statistical information are provided in Supplementary Data 1.

.

Figure S6

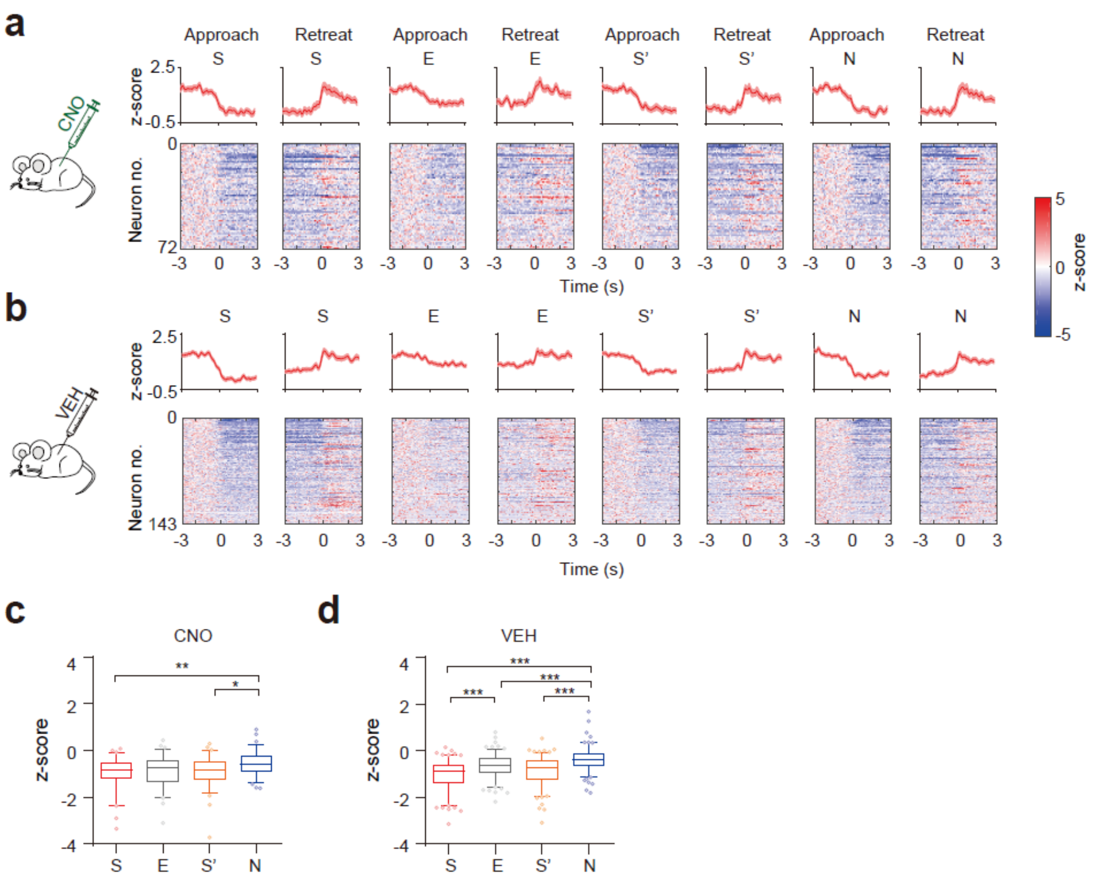

Supplementary Figure 6. mPFC neurons with suppressed responses during chemogenetic inhibitions of the Re

a, **b**, Response profiles of suppressed neurons to S, E, S' and N aligned at the beginning (approach) and at the end (retreat) of the investigation when under the treatment of CNO of VEH in mice infected with AAV-hSyn-hM4D-mCherry.

c, **d**, Average response for each stimulus. Kruskal-Wallis test with Dunn's multiple comparison correction. **c**, n = 72 for S, E, S', N. $H(3) = 14.66$, $P = 0.0021$ **d**, n = 143 for S, E, S', N. $H(3) = 88.97$, $P < 0.0001$.

*$P < 0.05$, **$P < 0.01$, ***$P < 0.001$. Box plots showing median, 25%-75% percentile, 5%-95% range and outliers. Line and shaded areas are mean ± s.e.m. Details of the statistical information are provided in Supplementary Data 1.

Figure S7

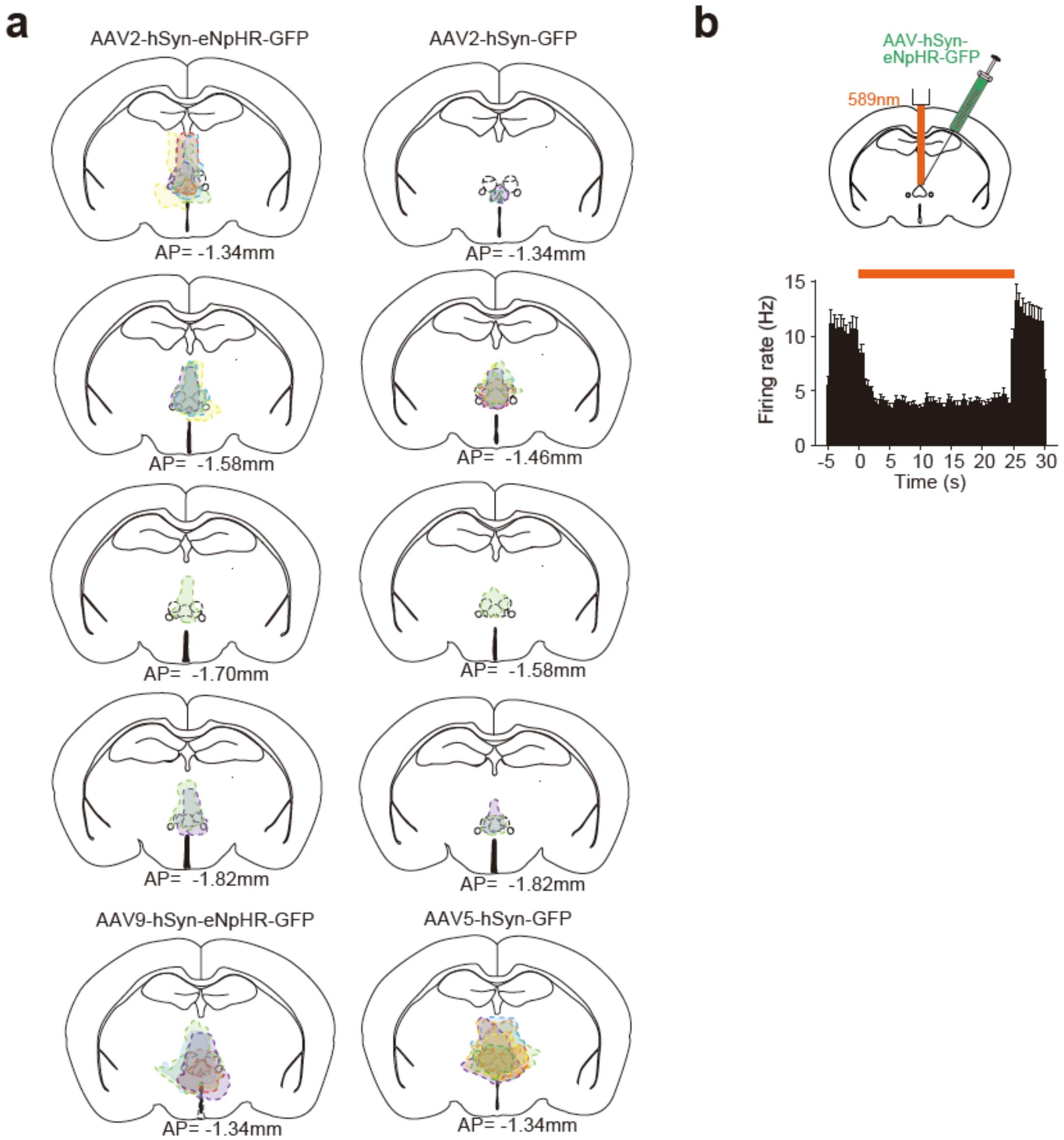

Supplementary Figure 7. Optogenetic virus expression

a, Schematic drawing showing the AAV-hSyn-eNpHR-GFP and AAV-hSyn-GFP expressions in the Re. Each colored area corresponds to the virus expression in one mouse. Virus serotypes were indicated. The coronal brain atlas was produced based on Allen Brain Atlas from https://atlas.brain-map.org/.

b, Peri-stimulus time histogram (PSTH) of single-unit activities recorded by the *in vivo* optrode recordings showing that yellow light effectively inhibited Re neural activities.

Figure S8

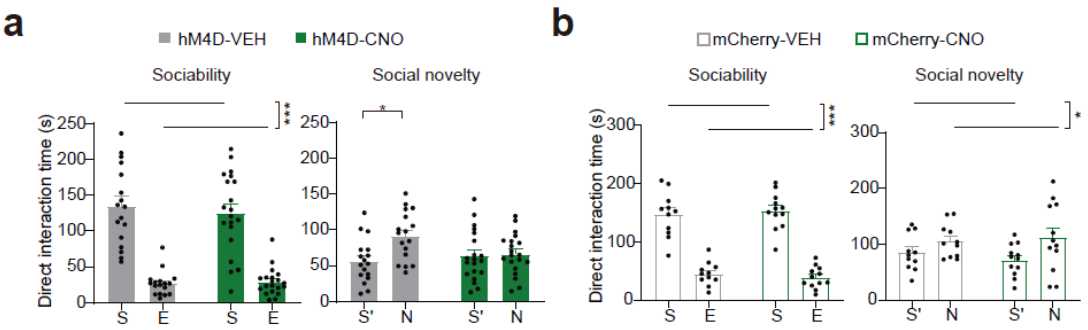

Supplementary Figure 8. Social behavior during chemogenetic inhibitions of the Re

a, b, Time spent investigating the stimuli in the three-chamber tests in mice expressing AAV-hSyn-hM4D-mCherry (**a**) and AAV-hSyn-mCherry (**b**). **a**, VEH: $n = 17$ mice, CNO: $n = 20$ mice. Sociability: stimulus $F(1, 70) = 112.7$, $P < 0.0001$; Social novelty: stimulus × treatment $F(1, 70) = 3.597$, $P = 0.062$. **b**, VEH: $n = 11$ mice, CNO: $n = 12$ mice. Sociability: stimulus $F(1, 42) = 166.7$, $P < 0.0001$; Social Novelty: stimulus $F(1, 40) = 6.254$, $P = 0.0164$.

Two-way ANOVA with Bonferroni correction. $^*P < 0.05$, $^{***}P < 0.001$. Data are mean ± s.e.m. Details of the statistical information are provided in Supplementary Data 1.
