# Supplementary material for: A prefrontal-thalamic circuit encodes social information for social recognition
Source: Nat Commun. 2024 Feb 3;15:1036. doi: 10.1038/s41467-024-45376-y (PMC10838311; doi:10.1038/s41467-024-45376-y)
Supplement: Supplementary file 3 — Description of Additional Supplementary Files [file 41467_2024_45376_MOESM3_ESM.pdf]

Description of Additional Supplementary Files

Supplementary Data 1 - Supplementary data for statistical analysis
